# HybridWeaveNet: deep cultural pattern recognition for Indian handloom heritage fabrics

**DOI:** 10.3389/frai.2026.1809586

**Published:** 2026-05-28

**Authors:** S. A. Anuraag Shankar, A. Sasithradevi, G. Krishnaraj, S. Kanimozhi

**Affiliations:** 1 School of Computer Science and Engineering, VIT University, Chennai, India; 2 Centre for Advanced Data Science, VIT University, Chennai, India; 3 Textile Design Department, National Institute of Fashion Technology, Chennai, India

**Keywords:** attention mechanisms in computer vision, deep learning-based fabric classification, EfficientNetV2-based feature learning, fine-grained pattern recognition, heritage textile analysis

## Abstract

**Introduction:**

India’s handloom sector represents the diversity of local culture, which is reflected in the traditional handwoven products from India. Regional fabric varieties such as Bandhani, Banarasi, Kancheepuram, Patola and Tussar are characterized by unique pattern structures, in terms of yarns as well as color arrangements. Accurate identification between them is crucial for tasks such as digital archiving, automatic categorization of fabric and e-commerce. Manually categorizing these designs would be tedious and subjective.

**Methods:**

Further, the existing conventional deep learning and computer vision approaches encounter significant challenges when processing complex motifs, fine-grained textures and substantial intra-class variability. For this purpose, we propose HybridWeaveNet, a custom deep learning architecture specifically developed to mitigate these shortcomings. The model integrates both a Dual Attention mechanism and pretrained EfficientNetV2 backbone to enhance pattern recognition and variate feature learning. Training of the proposed model is carried out using balanced sampling among the available five-class in the dataset. Further, various augmentation methods, including Mixup, Cutmix, GridDropout, Cutout, Blur, and ColorJitter, have been employed for effective generalization.

**Results:**

The proposed HybridWeaveNet model is evaluated using various metrics such as accuracy, macro F1-score, Cohen’s kappa, Matthews correlation coefficient (MCC), Jaccard index and log loss and exhibited 91% performance.

**Discussion:**

Grad-CAM visualizations validated that the model concentrates on culturally related motifs, thereby highlighting its potential for extensive fabric classification.

## 1 Introduction

The Indian handloom tradition is profoundly embedded by cultural and religious frameworks. Further, they are served as a tool for expressing social identity in addition to its functionality. It reveals the craftmanship and social values in terms of lifestyle environment of the specific region. In this study, the traditional handloom products such as Bandhani, Patola, Banarasi and Kancheepuram are considered for the automated identification along with Tussar, a fabric derived from wild silk. The aforementioned products were selected as they contribute the society in numerous ways like cultural identity and providing employment opportunity.

Kancheepuram sarees are known for their rich texture, vibrant colors and distinctive traditional motifs such as temple designs. They often feature pure gold zari, contrast borders, korvai work with extra warp and weft patterns, elaborate detailing and rich appearance make them magnificence appearance. Banarasi saree is known for its gold and silver work on silk where satin weave has been used as base. The saree is produced with brocade designs by using intricate floral and Mughal-style motifs. The fabric has a lot of metallic visual effects and reflecting royal elegance style. In the case of Tussar silk saree, it is produced with thick, heavy textured Tussar silk yarns which are generally produced with stripe designs, coarse texture and without border. In addition, Tussar sarees offer various subtle designs such as tribal patterns, floral and geometric patterns. Patolas are produced with double-ikat technique where various forms like dancing woman, elephant, parrot, floral, vegetable, Islamic architectural patterns are used. Bandhani is created on silk, cotton and woolen fabrics where dots are produced by using a small point or spot, tear drop shape or cowrie shell, square spots and round spots.

Traditionally, the documentation and preservation of these practices have relied upon ethnographic research, interviews with artists, manual photographic documentation for museum and archival holdings. Yet, these approaches continue to be arbitrary, laborious and challenging to scale, which restricts their use to scholars, curators and the general public. This has delayed advancements in domains such as digital preservation, historical documentation and E-commerce integration, owing to the absence of systematic and technologically advanced digitization methods.

Recent advances in artificial intelligence and computer vision offer promising alternatives (Selvaraju et al., 2020). While traditional computer vision tools can inspect surface details and detect fabric imperfections, they often fall short when analyzing the complex designs and intricate motifs present in Indian fabrics. Though Deep learning has a potential to process the visual data hierarchically, its application in rich handloom patterns remains limited. This could be due to lack of performance of CNN in detailed motif structures and intra-class variation (Woo et al., 2018; Baltrušaitis et al., 2018). It is most commonly occurred in traditional fabrics.

In this study, HybridWeaveNet, a specialized deep learning architecture is developed for classification of specific handloom varieties automatically based on their uniqueness. It is achieved by using a pretrained EfficientNetV2 backbone for extracting the unique characteristics such as motifs, surface variation by technique and color through Dual attention module. It improves the model ability to differentiate spatial and channel features. The training process is strengthened by using various regularization strategies like Mixup, Cutmix, label smoothing, and Exponential Moving Average (EMA).

The factors related to rotational symmetry, weave density, motif complexity have enabled computer vision to address the challenges of fabric and texture identification. The irregularity of handwoven fabrics continues to cause many of the current approaches to fall short, despite significant advancements in deep learning and hybrid approaches. In order to improve texture representation and reduce the number of parameters, Fujieda et al. (2018) developed Wavelet Convolutional Neural Networks, which incorporate Wavelet transforms into the composition of Convolutional Neural Networks. However, their method only takes into account a small number of crucial spatial correlations for identifying handloom motifs. Similarly, Qi et al. (2016) proposed the Local Orientation Adaptive Descriptor (LOAD), combining handcrafted descriptors with CNN-based features for rotation-robust classification, but its reliance on manually designed descriptors constrains adaptability to diverse fabrics. Marcos et al. (2017) further advanced texture alignment using rotation-equivariant CNNs with filter weight sharing, yet their method introduces computational overhead, making it less suitable for large-scale cultural fabric applications. Deep learning techniques (Han et al., 2015) have been explored in cultural fabric contexts outside India. Abd Manap et al. (2024) developed a CNN-based framework for classifying Indonesian and Malaysian batik patterns, leveraging MobileNet and YOLO architectures to achieve high accuracy.

Mahanta et al. (2024) used DenseNet-based architectures for differentiating handloom and powerloom fabrics. Iqbal Hussain et al. (2020) utilized the transfer learning to identify the patterns in woven fabrics. These aforementioned methods emphasized more on structure instead of identifying the subtle cultural differences in motifs. The other techniques such as graph-based learning (Sun et al., 2023) and capsule networks (Sabour et al., 2017) are designed to improve deformation resistance. However, as they are having limited adaptability and high processing requirements, these techniques are obstructed. Ozlem et al. (2024) integrated CNNs with statistical measures like GLCM and yielded significant outcome for Indian fabric classification. Yet, the cost of the model is increased with complexity. When compared to modern deep learning techniques, traditional feature descriptors such as fuzzy texture unit patterns frequently underperforming in capturing the erratic patterns of handloom fabrics.

In order to address the issues related to analysis of textiles, sustainability and the overall control of quality, nowadays the researches were moved toward artificial intelligence-based solutions. For example, the authors Akram et al. (2025) suggested a state-of-the-art fabric classification framework incorporating artificial intelligence for sustainable practices and the automated decision-making process in relation to textile product development. The primary focus of this work is to create a scalable and operationally efficient systems, rather than distinguishing between specific cultures or types of motifs on fabrics. In contrast, Mohammed et al. (2025) proposed a Fully Convolutional Neural Network (CNN) to enable quick defect detection, classification and segmentation of fabrics for Quality Inspections. As a result, they were able to achieve superior results in the area of quality inspections. However, this work focused on defect localization, rather than holistic fabric identity recognition (Wang et al., 2020).

Recently, Majumdar et al. (2025) perform a systematic review that encompassed deep learning applications within the textile sector. It includes advances in defect detection, classification of materials and optimization of processes. Mainly they focused on the absence of models that took cultural context into account when creating textile patterns (i.e., meaningful patterns). Butturi et al. (2025) have surveyed the contribution of intelligent systems toward enhancing the environmental goals. Also project the role of intelligent system that improves the innovation strategies which stimulate the recovery of pre-consumer textile waste and promote circular manufacturing. However, the work is limited by not investigating the use of these systems toward classification (Krizhevsky et al., 2017) for textile pattern-level images. These recent works provide insight regarding the need for AI on the environment, as well as highlights the knowledge gap regarding the classification of fine-grained images of traditional textiles between cultures. Table 1 provides the comprehensive survey that illustrates the limitations of existing fabric classification studies.

**Table 1:** Comprehensive summary of existing fabric classification.

S. no.	Author and paper	Problem addressed	Methodology	Limitation	HybridWeaveNet advantage
1	Almeida et al. (2021)	Defect detection in industrial fabrics	CNN + false negative reduction	Highly task-specific; limited applicability to culturally rich textile patterns	Learns discriminative cultural motifs using attention rather than defect localization
2	Wei et al. (2022)	Multi-scale texture recognition	Multi-scale CNN	Does not explicitly model semantic or spatial importance	Dual attention enhances both spatial relevance and channel importance
3	Zhou et al. (2023)	Pattern classification with limited data	ResNet-50 + augmentation	Sensitive to high intra-class visual variation	Robust training with Mixup, CutMix and EMA improves generalization
4	Sajitha and Priya (2024)	Subtle defect classification	Optimal artificial neural network-based fabric defect detection and classification (OANN-FDDC)	Designed primarily for defect analysis rather than semantic textures	Attention maps correspond to meaningful fabric motifs and structures
5	Hosny et al. (2021)	Deep + handcrafted features	Residual CNN + LBP	Relies on manual feature engineering and feature fusion	Fully end-to-end learning without handcrafted feature dependence
6	Mei et al. (2024)	Small defect detection	YOLOv8n-LAW + enhancement	Object-detection oriented; unsuitable for holistic texture classification	Global feature learning captures complete fabric structure
7	Aksakalli et al. (2025)	Complex defect types such as mixed defects	Hybrid PatchNet–attention mode	High model complexity and limited scalability	EfficientNetV2 backbone offers a better accuracy–efficiency trade-off
8	Tao et al. (2023)	Classify the fabric materials	GraphSAGE and bidirectional gated recurrent unit and layer attention mechanism (BiGRU-attention)	High computational and memory overhead	Lower computation based architecture enables easier deployment
9	Ramachandran et al. (2023)	Transformer-based defect detection	Hybrid ViT + ResNet-50 model	Data-hungry and computationally expensive	Performs well on moderate-sized datasets with lower computational cost
10	Sastypratiwi et al. (2024)	Batik pattern classification using TL	EfficientNet/MobileNet + Transfer Learning	Limited ability to handle high cultural and intra-class variability	Designed specifically for diverse and culturally rich handloom fabrics

Among Indian handloom varieties, Banarasi, Bandhani, Patola, Kanjeevaram and Tussar present distinct challenges during the automatic classification. Banarasi sarees, with their elaborate floral motifs and zari work, pose difficulties in feature encoding. Bandhani fabrics, characterized by tightly clustered tie-dye dots, introduce inconsistencies at the pixel level that complicate analysis. The geometric imperfection of double ikat weaving in Patola fabrics makes it difficult to recognize fine-grained designs. Kanjeevaram silks demand hue-invariant representations because of their sharp contrast colors, powerful temple-bordered motifs, and high contrast hues. Lastly, the significant intra-class similarity of earthy-toned, coarse-textured Tussar fabrics lessens separability.

The shortcomings stated above highlight the need for frameworks that can capture the details of localized motifs without relying too heavily on computationally costly handcrafted elements or designs. Hence, we present HybridWeaveNet, a domain-aware deep learning architecture that makes use of dual channel and spatial attention modules in addition to a pretrained EfficientNetV2 backbone. Previous initiatives concentrated on a solely handmade data generation model, which contrasted with the data-driven paradigm of HybridWeaveNet. We use significant data augmentations with other regularization methods including Mixup, Cutmix, Dropout, and Exponential Moving Average (EMA) (Shorten and Khoshgoftaar, 2019) to achieve better generalization while lowering overfitting. We show how our model may be used as a reference point for fine-grained classification of culturally significant Indian handlooms using a carefully selected five-class dataset that includes Banarasi, Bandhani, Kancheepuram, Patola, and Tussar fabrics.

The key contributions of this research include:

- Constructed of the Fabric WeaveNet Dataset: In the aim of developing a benchmark for cultural fabric classification, a high-resolution, well-balanced dataset is created by featuring five prominent types of Indian handloom fabrics.
- Hybrid Architecture: Introduction of a tailored framework, HybridWeaveNet, which integrates EfficientNetV2 with dual attention mechanisms to effectively distinguish intricate fabric motifs.
- Comprehensive Evaluation: A wide range of evaluation metrics along with interpretability tools such as Grad-CAM and calibration plots, were used to ensure clarity, adaptability and practical relevance of proposed prediction model.

## 2 Methods

**Figure 1:**
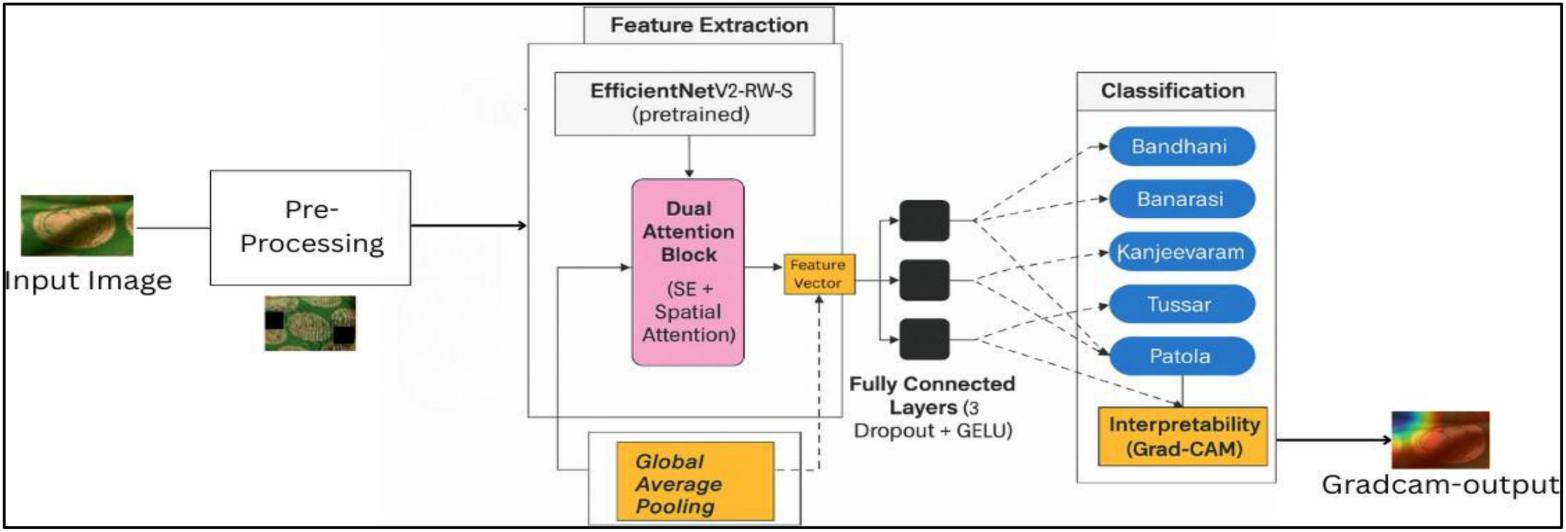
Overall workflow for Indian handloom fabric classification using HybridWeaveNet.

The proposed fabric pattern analysis model is carried out in three key stages such as preprocessing, model development and prediction as shown in Figure 1. Initially in preprocessing stages various augmentation techniques including resizing, flipping, color-jitter, griddropout, cutout was performed to expose the model toward diverse pattern variation and to avoid overfitting. During training, a class-weighted sampling strategy was utilized to maintain a balanced classification among the fabric classes in the dataset.

Secondly, in the core model construction stage, the HybridWeaveNet, is built on the EfficientNetV2-RW-S backbone, which includes more than 30 layers. The backbone is integrated with dual attention modules to capture both spatial and channel-level feature relationships. The training workflow incorporates with several techniques such as mixup, cutmix, label smoothing, Exponential Moving Average (EMA) as well as a warmup-cosine learning rate scheduler for smoother optimization and stronger generalization. A dense classification head is also equipped with dropout, improves regularization and reduce overfitting during training. These techniques collectively support stable and efficient convergence.

Finally, during the prediction stage, the model processes unlabelled fabric images, produce its class labels and generates Grad-CAM visualizations. These activation maps visually highlight the most influential regions of the image, enhancing the interpretability of the model’s decisions. This is particularly valuable for applications in digital fabric commerce, the preservation of cultural heritage and enhances the transparency of automated recognition systems.

### 2.1 Preprocessing pipeline

The dataset comprises high-resolution images of fabrics, which undergo a series of augmentations aimed at improving model generalization and minimizing overfitting. These augmentations include resizing, horizontal flips, color jittering, GridDropout, Cutout, random brightness and contrast adjustments, blurring, scaling, rotation, and spatial shifting. Geometric operations like rotation and scaling are applied through affine transformations, described by Equation 1:


$$T(x,y)=[\begin{array}{c} s\cdot \cos\theta -s\cdot \sin\theta \;t_{x}\\ s\cdot \sin\theta \;s\cdot \cos\theta \;t_{y} \end{array}][\begin{array}{c} x\\ y\\ 1 \end{array}]$$ (1)

where *s* denotes the scaling factor, θ is the rotation angle and $t_{x}$ , $t_{y}$ are translation offsets.

GridDropout and Cutout act as regularization mechanisms by randomly obscuring portions of the image. This forces the model to develop more resilient global feature representations. Their impact is mathematically expressed in Equation 2 using a binary mask:


I˜(x,y)=I(x,y)·M(x,y),M(x,y)∼Bernoulli(1−p) (2)

To achieve balanced learning across fabric categories, we apply a weighted sampling approach on the stratified dataset. Each class i is assigned a weight inversely related to its frequency, as shown in Equation 3:


$$W_{i}=\frac{1}{f_{i}}$$ (3)

where $f_{i}$ denotes the frequency of samples in class *i*.

### 2.2 Model architecture: HybridWeaveNet

**Figure 2:**
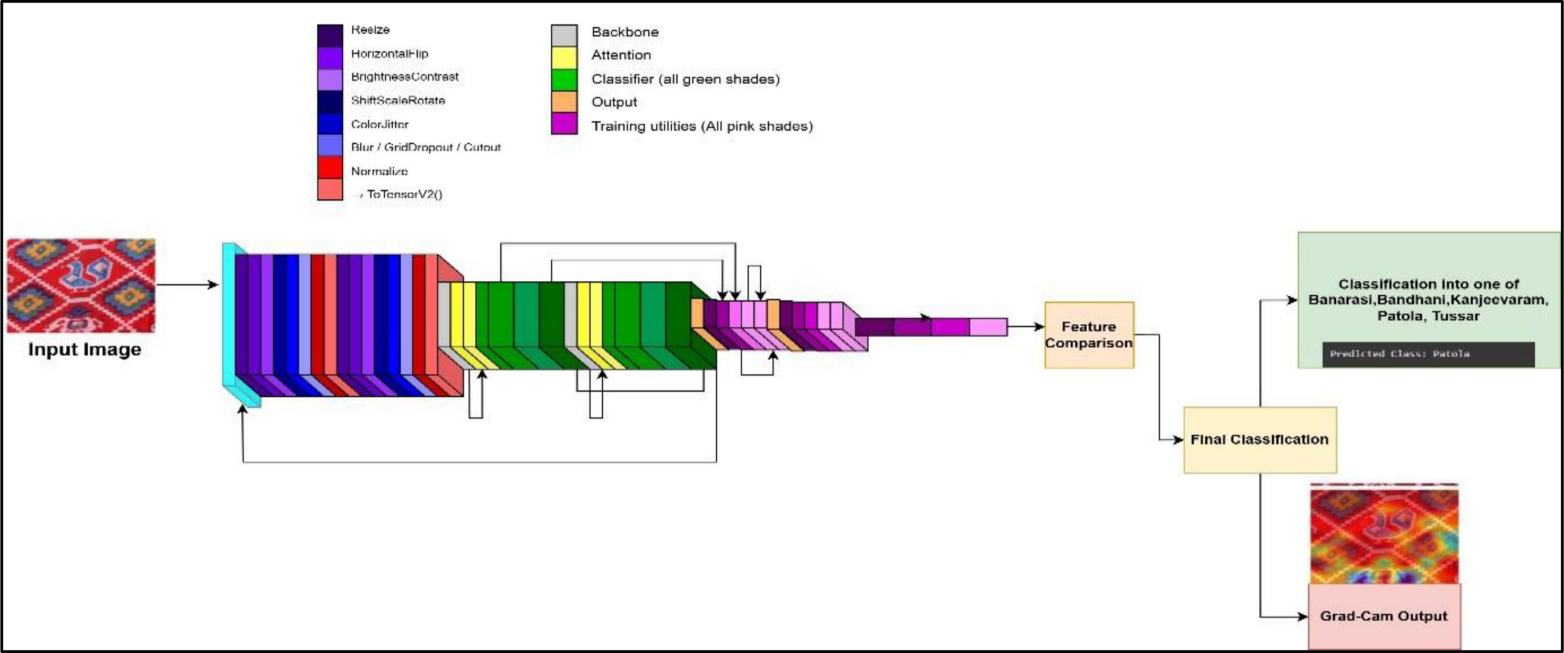
HybridWeaveNet overall architecture with backbone, dual attention blocks, and classification head.

The proposed model, HybridWeaveNet, is designed to capture fine-grained structural cues and motif-specific details inherent to Indian handloom fabrics shown in Figure 2. At its core, the network leverages EfficientNetV2-RW-S as the backbone, chosen for its parameter efficiency, ability to scale depth and width while maintaining computational feasibility. The backbone generates hierarchical feature maps, which are then refined by a dual attention block consisting of channel and spatial attention modules.

To emphasize discriminative feature channels, we integrate a squeeze-and-excitation mechanism defined as:


$$\mathrm{SE}(X)=\sigma (W_{2}\cdot \delta (W_{1}\cdot \mathrm{GAP}(X)))$$ (4)

where Equation 4, X is the input feature map, GAP(·) denotes global average pooling, $W_{1}$ and $W_{2}$ are learnable weights, δ is ReLU, and σ is Sigmoid. This operation adaptively reweights channels according to global context.

Complementary to channel weighting, spatial attention identifies key motif locations by combining average-pooled and max-pooled maps across channels:


$$M_{s}(X)=\sigma (f^{7\times 7}([\text{Avgpool}(X);\text{Maxpool}(X)]))$$ (5)

where in Equation 5, $f^{7\times 7}$ is a convolutional filter, and concatenation [·;·] integrates pooled descriptors. The refined representation is passed through Global Average Pooling (GAP) and a dense classifier comprising three fully connected layers with GELU activation and dropout regularization. The novelty of the proposed model lies in integrating the EfficientNetV2 layers based on capturing fine grained motif pattern and analyzing their structure discrimination with the help of attention mechanism. The final softmax layer predicts one of the five fabric categories. An overview of the architecture, including dual attention integration, is illustrated in Figure 2.

ALGORITHM 1 HybridWeaveNet training pipeline
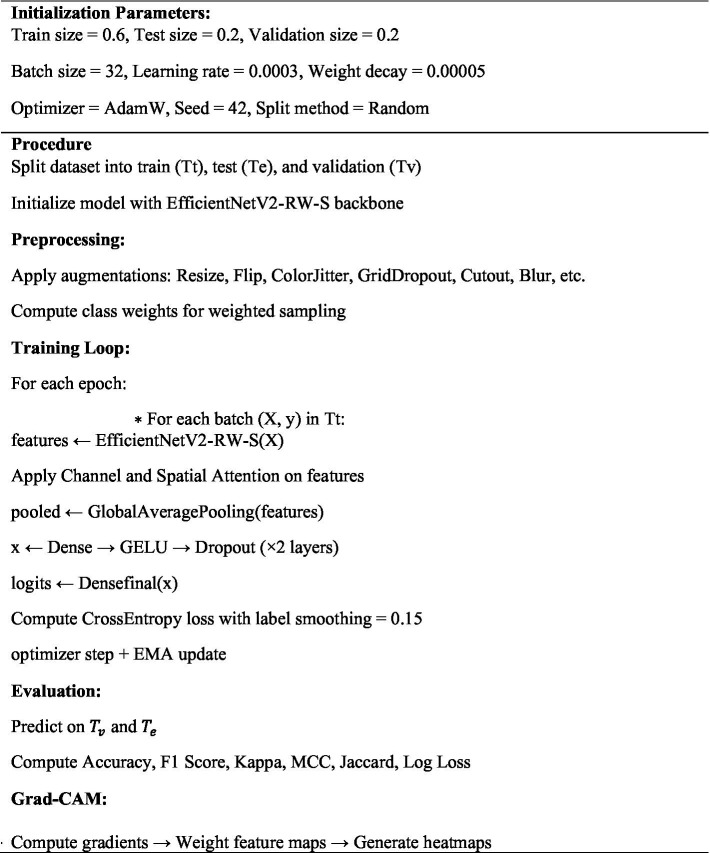

Algorithm 1 illustrates the essential initialization parameters and the sequential training process implemented for HybridWeaveNet. The dataset was randomly splits into three segments such as 60% for training, 20% for validation and 20% for testing. The training procedure used the AdamW optimizer with a low learning rate and weight decay to maintain stability. A batch size of 32 was selected to optimize computational efficiency. The backbone network was responsible for feature extraction, which was then enhanced through dual attention mechanisms before being passed through pooling layers and dense layers with dropout for regularization. Label smoothing and EMA were applied to improve robustness. Evaluation used multiple metrics and Grad-CAM visualizations enhanced interpretability.

**Figure 3:**
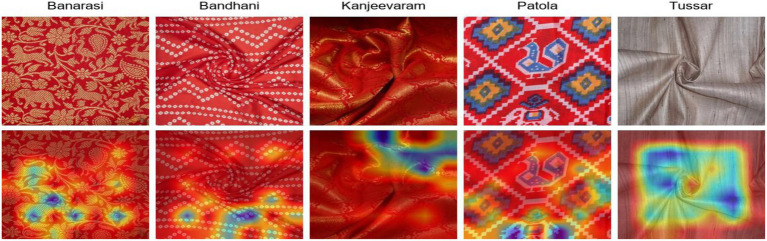
Grad-CAM overlays highlighting class-discriminative regions for Banarasi, Bandhani, Kanjeevaram, Patola, and Tussar fabric samples.

In the inference stage, unseen fabric images are processed through the backbone, attention blocks and classifier to yield fabric class probabilities. To improve interpretability, we adopt Grad-CAM (Gradient-weighted Class Activation Mapping), where discriminative weights for class c and feature channel k are computed as:


$$a_{k}^{c}=\frac{1}{Z}\sum_{i}\sum_{j}\frac{\partial y^{C}}{\partial A_{\mathrm{ij}}^{K}}$$ (6)

here in Equation 6, $A^{K}$ is the activation map of channel k , $y^{C}$ is the score for class c , and Z is the spatial resolution. The final Grad-CAM map highlights the regions most responsible for the prediction, enabling transparent validation of model decisions which is shown in Figure 3.

Developing a reliable model to identify handloom fabrics is highly challenging. The creation of a balanced dataset was time-consuming and imposed substantial human review to eliminate duplicates and mislabeled items. This is necessary due to the large range of variations within a single class, including motifs, patterns, styles, weaves and color. Certain fabric types were also difficult to reliably categorize due to their close similarities, which is why the more complex discriminative model was needed for the simpler backbone baseline CNNs. The only optimization challenge was manually adjusting learning rate schedules to mitigate the effects of aggressive augmentation techniques like mixed cutmix and mixup, which distorted the motifs. EfficientNetV2-RW-S’s predictions with attention blocks best satisfied the necessary balance between precision and computational cost. The fabric classification using HybridWeaveNet architecture is trained initially for 50 epochs but executed with early stop for 32 epoch to avoid overfitting. Table 2 provides the detailed information on developed architecture with 80% is used for training and 20% is reserved for testing.

**Table 2:** Hyper-parameter settings and architectural specification of HybridWeaveNet.

Component	Specification
Training hyper-parameters	
Optimizer	AdamW
Learning rate	0.0003
Loss function	Categorical cross-entropy (label smoothing = 0.15)
Batch size	32
Weight decay	0.00005
Learning rate schedule	Warmup + Cosine annealing
Regularization	Dropout, Mixup, CutMix, EMA
Input configuration	
Input image size	224 × 224 × 3 (RGB)
Data augmentation	Flip, ColorJitter, Blur, GridDropout, Cutout
Backbone network	
Base architecture	EfficientNetV2-RW-S (ImageNet pretrained)
Feature extraction depth	∼30 convolutional layers
Attention modules	
Channel attention	Squeeze-and-excitation (SE)
Spatial attention	Convolution-based spatial attention (7 × 7)
Dual attention placement	After final convolutional block
Feature aggregation	
Global pooling	Global average pooling (GAP)
Flattened feature size	1,280 (EfficientNetV2 output)
Classification head	
Fully connected layer 1	1280 → 512 (GELU)
Dropout rate	0.5
Fully connected layer 2	512 → 256 (GELU)
Dropout rate	0.3
Output layer	256 → 5 (Softmax)
Prediction and interpretability	
Test-time augmentation	Horizontal flip, multi-crop
Visualization	Grad-CAM

The proposed work is limited by a smaller dataset size of 2000 images and constrained diversity in data collection which includes the lighting conditions, viewpoints and backgrounds. Meanwhile the augmentation is applied to balance the dataset, it could not meet-up real-world data. Additionally, the dataset covers only five fabric classes, with high intra-class variation and inter-class similarity, which may affect generalization.

## 3 Results

### 3.1 Dataset overview and implementation details

The dataset used in this project is made up of carefully selected and current high-quality digital store photos of Indian handloom fabrics. The photos are manually checked for quality and balance. The five representative fabric types: Banarasi, Bandhani, Kanjeevaram, Patola, and Tussar, were examined in terms of their weaving styles, cultures and histories as shown in Table 3. Varying textural and structural characteristics across each class in the dataset enables fine-grained visual recognition. The proposed dataset not only supports the computational study, also provides digital preservation and documentation of traditional Indian fabrics.

**Table 3:** Dataset summary of Indian handloom fabric classes.

Class name	Image count	Image size (HxW)	Train/Val/Test split	Uniqueness of pattern
Banarasi	400	224 × 224	320/60/20	Intricate brocade with zari and floral motifs
Bandhani	400	224 × 224	320/60/20	Dot-based resist tie-dye with clustered patterns
Kanjeevaram	400	224 × 224	320/60/20	Thick silk weave with temple borders and contrast
Patola	400	224 × 224	320/60/20	Double ikat weave with geometric symmetry
Tussar	400	224 × 224	320/60/20	Coarse texture with earthy tones and irregular weave

The proposed HybridWeaveNet is built on the EfficientNetV2-RW-S backbone with dual-attention modules, maintaining a balance between accuracy and efficiency as illustrate in Table 4.

**Table 4:** Model complexity analysis.

Metric	Value
Total parameters	~24.6 million
Model size	~96 MB (FP32)
FLOPs	~9.2 GFLOPs (224 × 224 input)
GPU inference time	~18–25 ms/image
CPU inference time	~120–180 ms/image

Most parameters originate from the backbone, while attention modules add minimal overhead (~2.5 M parameters). Experiments were conducted on a CUDA-enabled GPU (e.g., NVIDIA RTX-class) with a batch size of 32. The model demonstrates efficient inference and a favorable trade-off between accuracy and computational cost, making it suitable for real-world deployment.

### 3.2 Overall performance metrics

Effectiveness of proposed HybridWeaveNet framework is evaluated extensively across all five fabric categories. In addition to calculating overall accuracy, the study performed class specific metrics such as precision, recall and *F*1-score. To measure the stability of decision boundary, the Cohen’s Kappa, Matthews Correlation Coefficient (MCC), were used. Similarly, to examine the set-level similarity between actual and predicted labels the Jaccard Index is employed. Also log loss evaluation is performed, ensuring the model produced well-calibrated predictions.

Accuracy: Equation 7 provides overall view of model performance across all fabric classes including Banarasi, Bandhani, Kanjeevaram, Patola and Tussar where true positives (TP) represent correctly classified fabric samples, true negatives (TN) denote correctly rejected non-class samples, false predictions are captured through false positive (FP) and false negative (FN) across all classes.


$$\text{Accuracy}=\frac{{\mathrm{TP}}_{\mathrm{fab}}+{\mathrm{TN}}_{\mathrm{fab}}}{{\mathrm{TP}}_{\mathrm{fab}}+{\mathrm{TN}}_{\mathrm{fab}}+{\mathrm{FP}}_{\mathrm{fab}}+{\mathrm{FN}}_{\mathrm{fab}}}$$ (7)

Precision: Equation 8 used to indicate the reliability of class specific prediction such as Kanjeevaram or Tussar.


$$\text{Precision}=\frac{{\mathrm{TP}}_{\mathrm{fab}}}{{\mathrm{TP}}_{\mathrm{fab}}+{\mathrm{FP}}_{\mathrm{fab}}}$$ (8)

Recall: Equation 9 to identify all true instance of fabric class, e.g. detecting true Patola cases.


$$\text{Recall}=\frac{{\mathrm{TP}}_{\mathrm{fab}}}{{\mathrm{TP}}_{\mathrm{fab}}+{\mathrm{FN}}_{\mathrm{fab}}}$$ (9)

*F*1-Score: Equation 10 to maintain the balance among the precision and recall as single measure.


$$F_{1}=2\times \frac{\text{Precision}\times \text{Recall}}{\text{Precision}+\text{Recall}}$$ (10)

Cohen’s kappa (*κ*): Equation 11 quantifies the reliability of model differentiating ability among the visually similar fabrics.


$$\kappa =\frac{p_{o}-p_{e}}{1-p_{e}}$$ (11)

where $p_{o}$ (observed agreement) is the proportion of instances where the predicted fabric class matches the true fabric class. $p_{e}$ (expected agreement) is the proportion of agreement expected to occur by random chance based on the class distribution.

Matthews Correlation Coefficient (MCC): Equation 12 a robust metric particularly useful in cases of class imbalance, such as with Tussar or Bandhani fabrics.


$$\mathrm{MCC}=\frac{{\mathrm{TP}}_{\mathrm{fab}}\times {\mathrm{TN}}_{\mathrm{fab}}-{\mathrm{FP}}_{\mathrm{fab}}\times {\mathrm{FN}}_{\mathrm{fab}}}{\sqrt{\begin{array}{l} \left({\mathrm{TP}}_{\mathrm{fab}}+{\mathrm{FP}}_{\mathrm{fab}})({\mathrm{TP}}_{\mathrm{fab}}+{\mathrm{FN}}_{\mathrm{fab}}\right)\\ \left({\mathrm{TN}}_{\mathrm{fab}}+{\mathrm{FP}}_{\mathrm{fab}})({\mathrm{TN}}_{\mathrm{fab}}+{\mathrm{FN}}_{\mathrm{fab}}\right) \end{array}}}$$ (12)

Jaccard Index (Macro): Equation 13 reflects the model ability to identify correct patterns among the overlapped actual and predicted results.


$$\text{Jaccard}=\frac{{\mathrm{TP}}_{\mathrm{fab}}}{{\mathrm{TP}}_{\mathrm{fab}}+{\mathrm{FP}}_{\mathrm{fab}}+{\mathrm{FN}}_{\mathrm{fab}}}$$ (13)

Log Loss (Cross-Entropy Loss): Equation 14 evaluates the confidence level of predicted probabilities across the five fabric types: Banarasi, Bandhani, Kanjeevaram, Patola, and Tussar.


$$\text{LogLoss}=-\frac{1}{N}\sum_{i=1}^{N}\sum_{j=1}^{C}y_{\mathrm{ij}}\log(p_{\mathrm{ij}})$$ (14)

where *N* represent total number of fabrics, *c* denotes no. of fabric class, $y_{\mathrm{ij}}$ is the ground truth label indicator and $p_{\mathrm{ij}}$ is the predicted probability that sample *i* belongs to fabric class *j*. Table 5 provides a summary of the model’s overall test performance along with challenges encountered across the five categories of handloom fabrics.

**Table 5:** Class-wise precision, recall, *F*1-score and challenges for HybridWeaveNet.

Class	Precision	Recall	*F*1-score	Challenges
Banarasi	0.89	0.80	0.84	Confusion with Kanjeevaram due to similar zari work and color patterns
Bandhani	0.95	0.90	0.92	Fine dotted patterns may overlap with textured fabrics
Kanjeevaram	0.86	0.90	0.88	High similarity with Banarasi in motifs and color composition
Patola	0.93	0.97	0.95	Minimal challenges due to distinct geometric patterns
Tussar	0.93	0.97	0.95	Slight variation in texture but generally well distinguished

The proposed HybridWeaveNet model achieves a test accuracy of 91.0%, which demonstrates the strong classification capabilities. Furthermore, the model’s consistency and balanced behavior is highlighted with obtained values of the Cohen’s Kappa (0.8875) and the Matthews Correlation Coefficient (MCC) (0.8879), even in the presence of differences among fabric classes. The model’s ability to accurately identify patterns in complex textures is reflected by a Jaccard Index of 0.8362. Additionally, the classifier stability and accuracy are projected well with the obtained low Log Loss score (0.4511).

Furthermore, Table 5 provides the additional details by displaying the class-specific results for precision, recall and *F*1-score. Among the five fabric types, patola and tussar achieved the highest *F*1 scores (0.95). Their unique geometric patterns and textural features, were effectively identified by the dual attention mechanism. Bandhani also showed strong results (*F*1 = 0.92), indicating that the model could clearly distinguish its intricate tie-dye patterns. In comparison, banarasi fabrics scored slightly lower in recall (0.80) and *F*1 (0.84), suggesting occasional confusion with similar silk weaves such as Kanjeevaram. Nevertheless, both the weighted and macro-average scores maintained a consistent value of 0.91, demonstrating balanced and reliable classification across all five fabric categories.

Table 6 provides the results obtained from the test set inputs. In this the high Cohen’s kappa and MCC describes the robustness and reliability across considered five classes. Similarly, the Jaccard index value reflects the overlap among the actual vs. predicted labels, with low log loss demonstrates well-calibrated probabilistic outputs.

**Table 6:** Overall performance metrics of HybridWeaveNet on the test-set.

Metric	Value
Test accuracy	91.0%
Cohen’s kappa	0.8875
Matthews correlation coefficient (MCC)	0.8879
Jaccard index (Macro)	0.8362
Log loss	0.4511

### 3.3 Visual evaluation and interpretability

**Figure 4:**
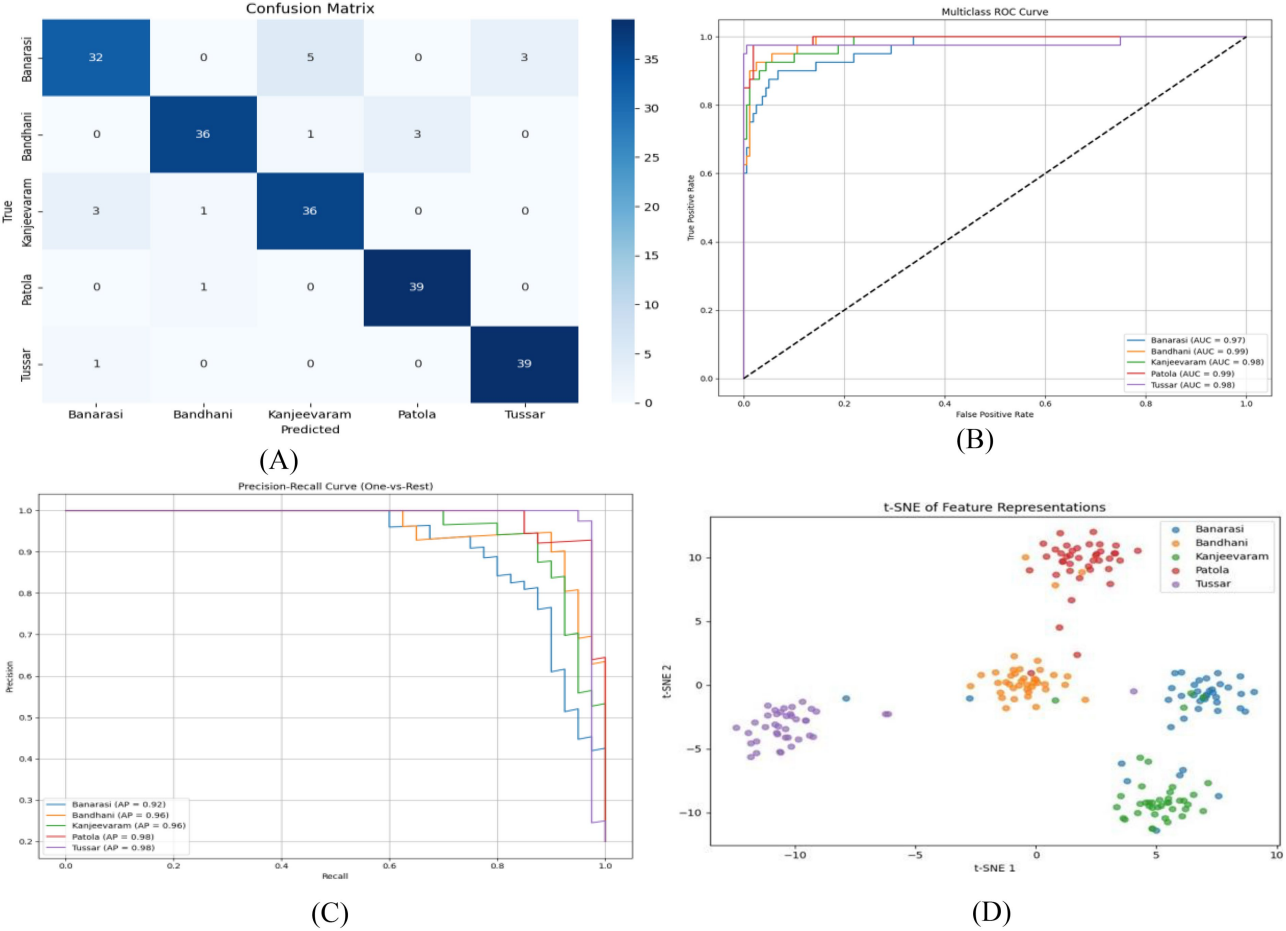
Comprehensive performance evaluation of HybridWeaveNet, including **(A)** confusion matrix, **(B)** multiclass ROC curves, **(C)** precision–recall curves and **(D)** t-SNE feature space visualization across Banarasi, Bandhani, Kanjeevaram, Patola and Tussar fabric.

Figure 4 provides an in-depth performance analysis of the HybridWeaveNet framework through a series of complementary evaluations. The confusion matrix (Figure 4A) illustrates the model’s strong classification performance across all fabric types, with dominant diagonal structure and minimal misclassification. Only a few misclassifications occur, predominantly between Banarasi and Kanjeevaram fabrics, which can be attributed to their closely related structural designs. The multiclass ROC curves shown in Figure 4B further emphasize the model’s high discriminative power.

Each fabric category achieved an area under the curve (AUC) score exceeding 0.97, reflecting excellent sensitivity, specificity and less false detection across a range of thresholds. Additionally, the precision recall curves in Figure 4C display precision values typically between 0.96 and 0.98. These results confirm the model’s reliability, even when challenged by class distribution imbalances and demonstrate its ability to maintain a consistent trade-off between recall and precision in fine-grained fabric classification.

Lastly, the t-SNE visualization in Figure 4D showcases how well the model separates learned features across different fabric types. Distinct and tightly grouped clusters appear for each category, with little overlap. This pattern indicates that the model effectively captures key discriminative characteristics, such as motif density, color variation and texture. Altogether, these evaluations support the HybridWeaveNet framework’s capability to perform accurate, interpretable and broadly applicable classification of Indian handloom fabrics.

### 3.4 Performance analysis of proposed model in analyzing the fabric pattern

**Figure 5:**
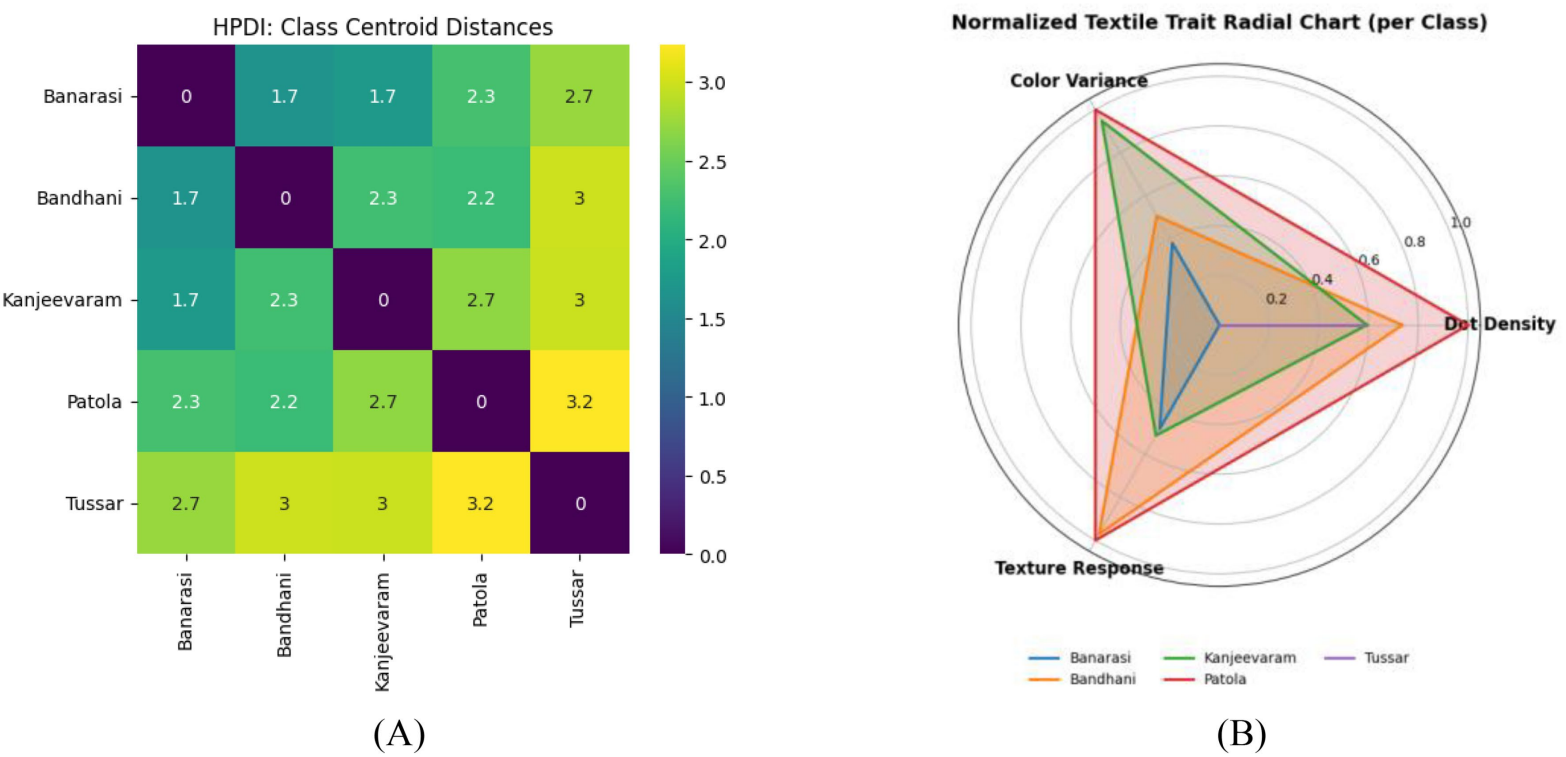
Illustrate the performance analysis, **(A)** Centroid distance heatmap, **(B)** radial fabric trait chart.

In order to give information on class separability and trait dominance for identifying handloom fabrics, Figure 5 integrates several representations. The distance matrix of centroids in the Highest Posterior Density Interval (HPDI) calculates the pairwise class inter-distinctiveness. It illustrates the near differences between Banarasi and Bandhani, which can be explained by their common motifs, but Patola and Tussar are the most distant and different. This is in line with the divergent structural patterns of the Patola and Tussar. The distribution of major discriminating traits is made clearer by the distance from the normalized radial chart’s center. Patola is the main class in terms of texture and reacts significantly to color changes, while Bandhani is characterized by dominant dot density. Kanjeevaram receives a balanced, reasonable score for every feature. To improve the understanding of the suggested categorization model, these data show the connection between the quantitative component of separability and the qualitative dominance of a trait. The quantitative evaluation across multiple dimensions provides us deeper insight into the discriminative capacity of the proposed framework.

Table 7 highlights intra-class similarity patterns, where Tussar displays the highest cohesion, suggesting relatively consistent visual traits, while Banarasi shows greater intra-class variability, showing diverse design details.

**Table 7:** CPSI: average intra-class similarity.

Class	Fabric type	Avg intra-class similarity
0	Banarasi	0.228998
1	Bandhani	0.366841
2	Kanjeevaram	0.356206
3	Patola	0.399266
4	Tussar	0.461017

**Figure 6:**
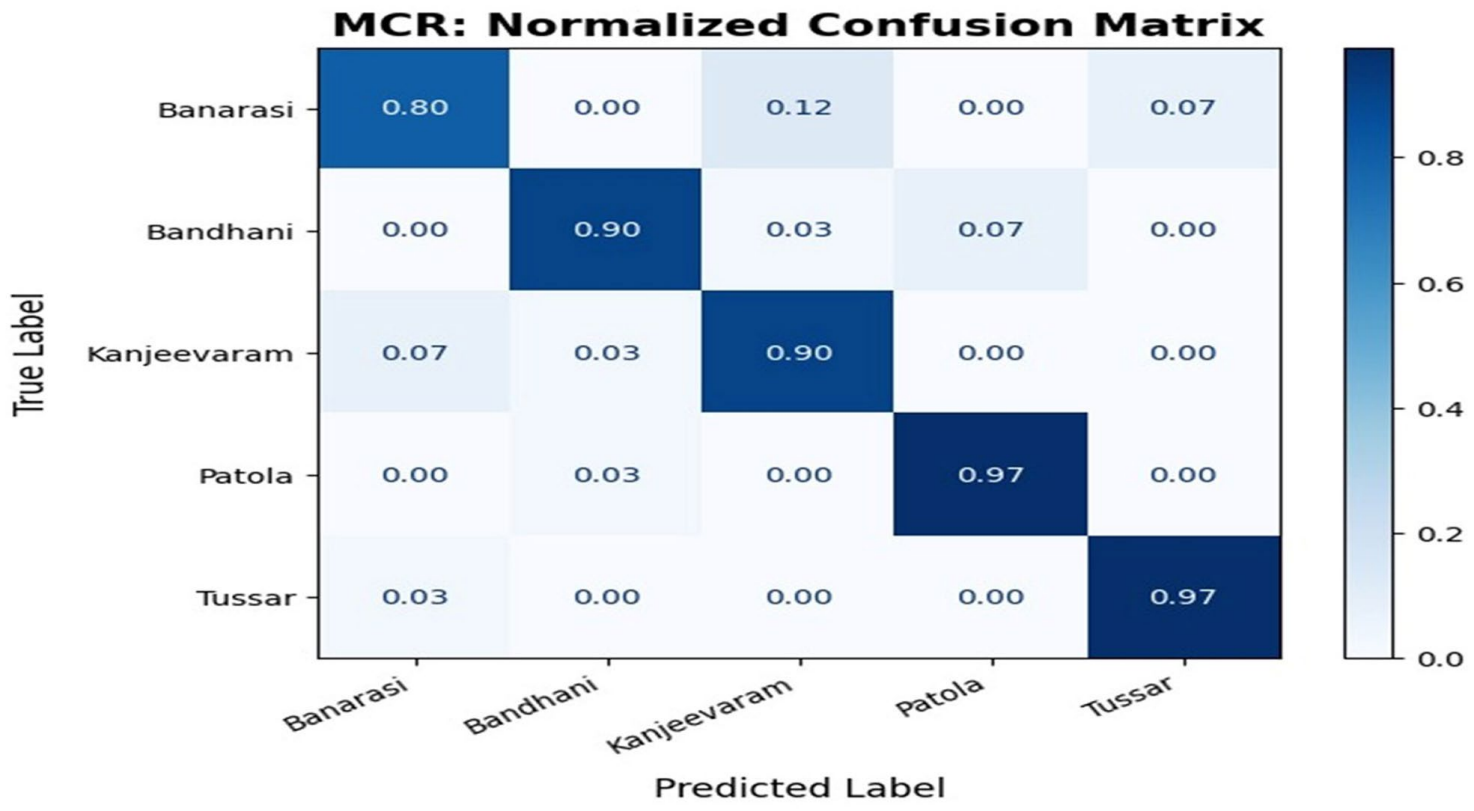
Visualization of classification accuracy across fabric classes.

The confusion matrix in Figure 6 demonstrates robust classification performance, with Bandhani, Patola, and Tussar achieving near-perfect recognition, whereas limited misclassifications occur between Banarasi and Kanjeevaram, indicative of overlapping ornamental features.

Table 8 shows the validation accuracy and validation loss at various representative training epochs for HybridWeaveNet. Within the first few epochs, the rapid growth of validation accuracy is accompanied by the continuous drop of validation loss, which suggests that effective feature learning is acquired. After 15–20 epochs, the validation accuracy starts to converge, indicating the model is converging while the validation loss remains at about 0.8. At epoch 32, the best performance on validation is achieved without noticeable further improvement. Thus, to avoid overfitting, early stopping is adopted to retain the best model for the final evaluation.

**Table 8:** Evaluation metric values across different epochs for HybridWeaveNet.

No. of epochs	Validation accuracy (%)	Validation loss
5	81.25	1.0737
10	87.25	0.8637
15	89.00	0.8210
20	89.25	0.8726
25	88.50	0.8273
30	89.50	0.8202
32	91.00	0.8081

Additionally, Table 9 provides a summary of patch-level (PLA) and variance-based grayscale statistics (VGS) used as texture descriptors. Among the fabric categories, Patola exhibits the highest entropy, indicating greater structural complexity, whereas Tussar shows lower entropy values, reflecting more uniform intensity patterns. These findings collectively highlight the model’s strength in capturing class-specific patterns, identifying subtle overlaps between similar fabric types and distinguishing them through detailed texture-based features. This reinforces both the interpretability and reliability of the model’s classification results.

**Table 9:** PLA/VGS: average patch texture stats per class.

Class	Fabric type	Mean	Std	Entropy
0	Banarasi	125.952073	48.074556	7.038893
1	Bandhani	124.633937	47.117453	6.844506
2	Kanjeevaram	128.846608	49.161081	6.983999
3	Patola	123.767498	48.027150	7.167961
4	Tussar	126.667574	45.179838	6.827542

### 3.5 Comparative study

**Figure 7:**
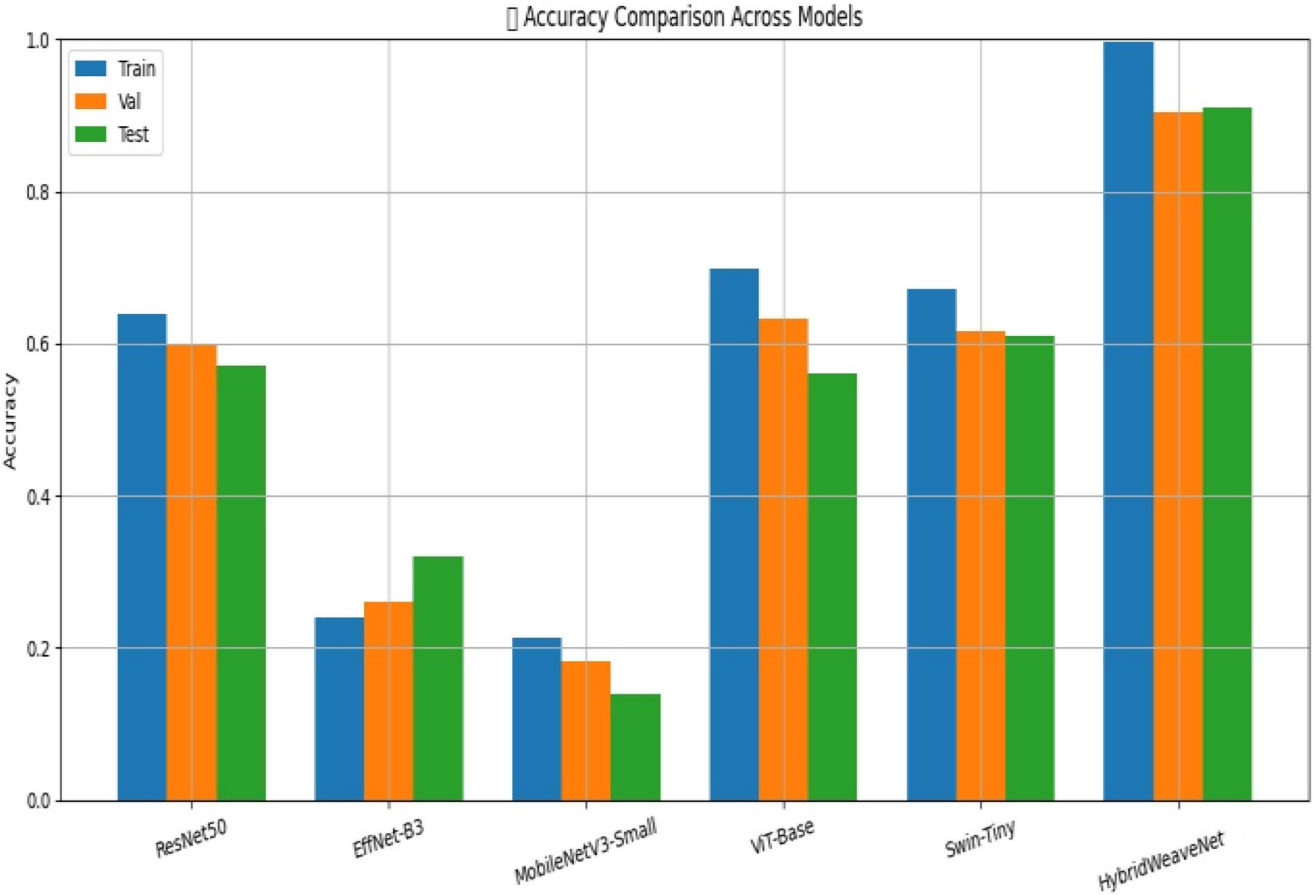
Comparison of validation accuracies between HybridWeaveNet and five pretrained models.

To evaluate the effectiveness of the proposed HybridWeaveNet model, a set of five popular pretrained architectures was selected: ResNet-50, EfficientNet-B3 (Tan and Le, 2021), MobileNetV3-Small, ViT-Base, and Swin-Tiny (Liu et al., 2021) illustrated in Figure 7. These models were chosen to represent a diverse range of architectural paradigms, including traditional convolutional networks (ResNet), scalable efficient models (EfficientNet), lightweight mobile-optimized networks (MobileNetV3) and transformer-based (Dosovitskiy et al., 2020; Khan et al., 2022) vision models (ViT and Swin).

**Figure 8:**
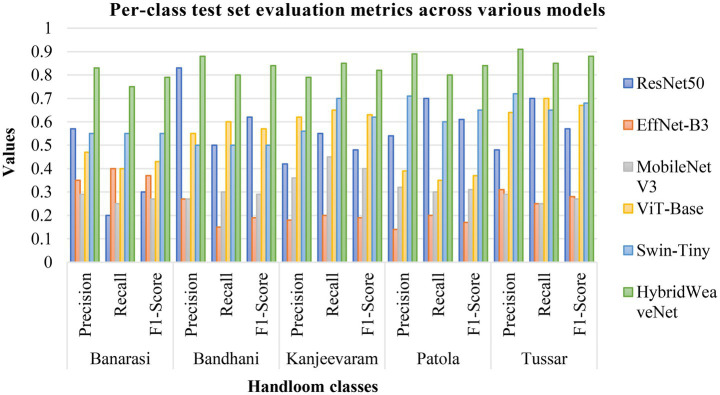
Class based evaluation along with baseline models.

All models were initialized with ImageNet-pretrained weights and then fine-tuned for five class Indian handloom fabric classification by replacing and training only the classification head. The backbone layers were kept frozen to focus the adaptation process on the head. Training is carried out under consistent conditions such as five epochs, identical data augmentation strategies and the same weighted sampling scheme to ensure fairness. While this setup does limit the full potential of the pretrained architectures, it provides a controlled benchmark to assess relative performance. As shown in Figure 7, HybridWeaveNet demonstrated higher accuracy, validating its architectural adaptability and effectiveness for culturally rich, texture-dense Indian fabric classification under constrained learning settings. Figure 8 represents a detailed class-wise comparison across popular models. It highlights HybridWeaveNet’s strong per-class performance, especially for challenging categories like Banarasi and Patola.

Similarly, the Table 10 provides the overall accuracy obtained on the test fabric dataset across different epochs when evaluated with the baseline models. The results include the mean along with the standard deviation (±) over random initializations. The low variance observed confirms the stability of the proposed HybridWeaveNet model.

**Table 10:** Comparative analysis of proposed model with baseline models across various epochs.

Epoch	HybridWeaveNet (Acc %)	ResNet50 (Acc %)	EffNet-B3 (Acc %)	MobileNetV3 (Acc %)	ViT-base (Acc %)	Swin-Tiny (Acc %)
5	81.25 ± 0.85	64.00 ± 1.19	24.00 ± 1.51	21.00 ± 1.39	70.00 ± 1.10	67.00 ± 1.02
10	87.25 ± 0.72	66.50 ± 1.12	25.50 ± 1.43	22.50 ± 1.33	72.50 ± 1.05	69. 00 ± 0.96
15	89.00 ± 0.65	68.00 ± 1.00	27. 00 ± 1.40	24.00 ± 1.30	74.00 ± 1.03	70.50 ± 0.89
20	89.25 ± 0.60	69.00 ± 0.94	28.50 ± 1.35	25.50 ± 1.26	75.50 ± 0.96	72.00 ± 0.86
25	88.50 ± 0.58	69.50 ± 0.92	30.00 ± 1.31	27.00 ± 1.21	77.00 ± 0.91	73.50 ± 0.79
30	89.50 ± 0.55	70.00 ± 0.83	31.00 ± 1.24	29.00 ± 1.14	78.50 ± 0.84	75.00 ± 0.74
32	91.00 ± 0.48	70.50 ± 0.80	32.00 ± 1.19	30.50 ± 1.11	80.00 ± 0. 79	76.00 ± 0.71

## 4 Ablation study

To evaluate the specific contributions of architectural and training components within HybridWeaveNet, a detailed ablation study was conducted. The process started with the base EfficientNetV2-RW-S model and incrementally introduced enhancements such as Dual Attention, GridDropout, Mixup/Cutmix, Warmup combined with Cosine Learning Rate scheduling and the SAM optimizer. Each configuration was trained for different epochs under consistent conditions, using the same train-validation split and augmentation strategies. This approach ensures a fair assessment of each component’s impact based on metrics like Accuracy, *F*1-score, Cohen’s Kappa, Matthews Correlation Coefficient (MCC), Jaccard Index, and Log Loss.

Table 11 outlines the performance results of each incremental modification, offering insights into how individual elements influence the model’s overall behavior. These results illustrate the accuracy variations across different epochs. The baseline EfficientNetV2-RW-S model recorded a respectable accuracy of 82.33%. Adding the Dual Attention module resulted in improvements across all performance metrics, validating the benefit of incorporating both spatial and channel-based attention.

**Table 11:** Ablation results across HybridWeaveNet component additions for various epochs.

Model component	Epoch	Accuracy (%)	*F*1 score	Kappa	MCC
EfficientNetV2 baseline	5	82.33	0.8216	0.7792	0.7795
	15	87.19	0.8382	0.8049	0.8053
	25	88.51	0.8511	0.8183	0.8185
	32	89.31	0.8589	0.8261	0.8264
+Dual attention	5	83.33	0.8323	0.7917	0.7924
	15	89.09	0.8571	0.8232	0.8235
	25	90.39	0.8694	0.8364	0.8371
	32	91.21	0.8781	0.8451	0.8454
+GridDropout	5	82.00	0.8179	0.7750	0.7757
	15	88.48	0.8444	0.8093	0.8103
	25	89.81	0.8556	0.8221	0.8225
	32	90.53	0.8631	0.8301	0.8306
+Mixup/CutMix	5	81.33	0.8114	0.7667	0.7672
	15	88.01	0.8411	0.8046	0.8052
	25	89.62	0.8541	0.8196	0.8202
	32	90.33	0.8611	0.8274	0.8282
+Warmup + Cosine LR	5	76.00	0.7534	0.7000	0.7013
	15	86.79	0.8356	0.7951	0.7961
	25	89.43	0.8522	0.8161	0.8171
	32	90.79	0.8651	0.8311	0.8321
+SAM optimizer	5	78.67	0.7778	0.7333	0.7382
	15	87.91	0.8459	0.8069	0.8081
	25	89.30	0.8561	0.8203	0.8211
	32	90.42	0.8639	0.8311	0.8310
HybridWeaveNet (all combined)	5	68.33	0.6759	0.6042	0.6109
	15	88.83	0.8471	0.8131	0.8147
	25	90.53	0.8652	0.8331	0.8343
	32	91.00	0.8721	0.8411	0.8419

Although Mixup and Cutmix contributed modest improvements likely due to the limited training duration, GridDropout helped strengthen generalization by addressing overfitting. The inclusion of Warmup with Cosine Learning Rate and the SAM optimizer did not yield immediate benefits within the constrained 5-epoch training window. However, these components are expected to show greater effectiveness over longer training periods.

In order to investigate early convergence behavior rather than final classification performance, all model versions in the ablation study were deliberately trained for just five epochs. As seen in Figure 8, the entire HybridWeaveNet shows relatively poorer accuracy (68.33%) under this limited training environment. Since the proposed architecture is deeper and includes dual attention methods require more optimizing steps to stabilize, this outcome is expected. Simpler model configurations, on the other hand, converge faster in the early epochs. HybridWeaveNet gets a significantly higher accuracy of 91.0% when the training period is increased to 50 epochs, indicating that its performance advantage becomes apparent once enough training iterations are given. Obtained results emphasize the importance of appropriate training for assessing deep hybrid networks by showing that ablation outcomes reflect convergence aspects rather than architectural effectiveness.

The proposed research presents a new deep learning framework, HybridWeaveNet that performs fine-grained classification of Indian handloom fabric. HybridWeaveNet is a deep learning framework that utilizes an EfficientNetV2-RW-S backbone with an assortment of attention modules in both channel and spatial dimensions. These modules enable the model to encodes abstract semantic features and local texture patterns that are essential for their specific discrimination. Using advanced data augmentation coupled with regularization processes improved generalization and classification performance across five fabric types: Banarasi, Bandhani, Kanjeevaram, Patola and Tussar. HybridWeaveNet framework produced classification accuracy with both efficiency and efficacy, to the extent it outperformed other models. Additionally, Grad-CAM visualizations provided feature interpretability by demonstrating where in the images the model was attending to make its predictions, allowing for the transparency required for practical applications.

Beyond addressing technical concerns, the framework can provide scalability in the documentation of material culture in the digitization of handloom fabrics, allowing for further digital documentation of this traditional material culture to be digitized. Automated classification of fabrics also holds practical implications for the advancement of everything from museum collections and online retail, to intelligent inventory methods, in addition to helping preserve the documentation of traditional fabrics for future generations.

The proposed research can be enhanced in the future by increasing the size of the dataset to include a wider range of regional fabrics and capturing images taken under varied imaging environment. Additionally, the use of a transformer-based architecture may help the model learn dependencies for long-range and complex weave structures. In addition to this, the application of few-shot or semi-supervised learning may alleviate the issues posed by limited annotated cultural data. The model may also be improved for application on edge devices by composing the model with compression methods, such as pruning or quantization that may allow for real-time user interaction on mobile devices or cultural kiosks. Ultimately, the mixed-mode analysis of visual data and associative sources such as artisan profiles or descriptions of the weaves will have implications for subsequent development of deeper, blended systems that are a part of cultural informatics and sustainable heritage preservation.

## Data Availability

The datasets presented in this study can be found in online repositories. The names of the repository/repositories and accession number(s) can be found below: Soundararajan, Kanimozhi; A., Sasithradevi; Shankar S. A., Anuraag; G., Krishnaraj (2026), “Fabric WeaveNet Dataset,” Mendeley Data, V2, doi: 10.17632/5yv96kyc78.2.

## References

[ref1] Abd Manap N. Xiao Xuan L. Kumar Singh K. Sheikh Akbari A. Putra A. (2024). Classification of Malaysian and Indonesian batik designs using deep learning models. J. Telecommun. Electron. Comput. Engineer. 16, 23–30. doi: 10.54554/jtec.2024.16.04.004

[ref2] Akram N. Butt R. A. Qureshi M. A. (2025). AI-driven fabric classification: real-time implementation for sustainable textile practices in industry 5.0. Mater. Res. Express 12:085501. doi: 10.1088/2053-1591/add98f

[ref3] Aksakalli I. K. Demir K. Sokmen O. (2025). A hybrid PatchNet-attention based deep learning architecture for multi-type fabric defect classification in textile manufacturing and quality control. Eng. Sci. Technol. Int. J. 72:102231. doi: 10.1016/j.jestch.2025.102231

[ref4] Almeida T. Moutinho F. Matos-Carvalho J. P. (2021). Fabric defect detection with deep learning and false negative reduction. IEEE Access 9, 81936–81945. doi: 10.1109/access.2021.3086028

[ref5] Baltrušaitis T. Ahuja C. Morency L. P. (2018). Multimodal machine learning: a survey and taxonomy. IEEE Trans. Pattern Anal. Mach. Intell. 41, 423–443. doi: 10.1109/TPAMI.2018.2798607, 29994351

[ref6] Butturi M. A. Neri A. Mercalli F. Gamberini R. (2025). Sustainability-oriented innovation in the textile manufacturing industry: pre-consumer waste recovery and circular patterns. Environments 12:82. doi: 10.3390/environments12030082

[ref7] Dosovitskiy A. Beyer L. Kolesnikov A. Weissenborn D. Zhai X. Unterthiner T. . (2020). An image is worth 16x16 words: transformers for image recognition at scale. Available online at: https://arxiv.org/abs/2010.11929 (Accessed June 3, 2021)

[ref8] Fujieda S. Takayama K. Hachisuka T. (2018). Wavelet convolutional neural networks. Available online at: https://arxiv.org/abs/1805.08620 (Accessed May 20, 2018)

[ref9] Han S. Mao H. Dally W. J. (2015). Deep compression: compressing deep neural networks with pruning, trained quantization and Huffman coding. Available online at: https://arxiv.org/abs/1510.00149 (Accessed February 15, 2016)

[ref10] Hosny K. M. Magdy T. Lashin N. A. Apostolidis K. Papakostas G. A. (2021). Refined color texture classification using CNN and local binary pattern. Math. Probl. Eng. 2021:5567489. doi: 10.1155/2021/5567489

[ref11] Iqbal Hussain M. A. Khan B. Wang Z. Ding S. (2020). Woven fabric pattern recognition and classification based on deep convolutional neural networks. Electronics 9:1048. doi: 10.3390/electronics9061048

[ref12] Khan S. Naseer M. Hayat M. Zamir S. W. Khan F. S. Shah M. (2022). Transformers in vision: a survey. ACM Comput. Surv. 54, 1–41. doi: 10.1145/3505244

[ref13] Krizhevsky A. Sutskever I. Hinton G. E. (2017). ImageNet classification with deep convolutional neural networks. Communications of the ACM. 60, 84–90. doi: 10.1145/3065386

[ref14] Liu Z. Lin Y. Cao Y. Hu H. Wei Y. Zhang Z. . (2021). “Swin transformer: hierarchical vision transformer using shifted windows,” in Proceedings of the IEEE/CVF International Conference on Computer Vision, (New York, NY: IEEE), 10012–10022.

[ref15] Mahanta L. B. Mahanta D. R. Rahman T. Chakraborty C. (2024). Handloomed fabrics recognition with deep learning. Sci. Rep. 14:7974. doi: 10.1038/s41598-024-58750-z, 38575749 PMC10994934

[ref16] Majumdar A. Bhattacharyya R. Yadav V. S. (2025). Deep learning applications in textile industry: a systematic review of current status and delineating future research agenda. Arch. Comput. Methods Eng. 33, 2205, 1–2223. doi: 10.1007/s11831-025-10366-w

[ref17] Marcos D. Volpi M. Komodakis N. Tuia D. (2017) Rotation equivariant vector field networks. In Proceedings of the 2017 IEEE International Conference on Computer Vision (pp. 5048–5057). New York, NY: IEEE

[ref18] Mei S. Shi Y. Gao H. Tang L. (2024). Research on fabric defect detection algorithm based on improved YOLOv8n algorithm. Electronics 13:2009. doi: 10.3390/electronics13112009

[ref19] Mohammed S. S. Clarke H. G. Mahmood S. N. (2025). A fully convolutional neural network for fast detection, classification, and segmentation of fabric defects. Neural Comput. & Applic. 37, 23249–23272. doi: 10.1007/s00521-025-11495-w

[ref20] Ozlem K. Atalay A. T. Atalay O. Ince G. (2024). FogETex: fog computing framework for electronic textile applications. IEEE Internet Things J. 12, 6856–6874. doi: 10.1109/JIOT.2024.3490981

[ref21] Qi X. Zhao G. Shen L. Li Q. Pietikäinen M. (2016). LOAD: local orientation adaptive descriptor for texture and material classification. Neurocomputing 184, 28–35. doi: 10.1016/j.neucom.2015.07.142

[ref22] Ramachandran V. Madhavan R. Anand P. Vishanth A. Pradeep K. V. (2023). Improved fabric defect detection using a vision transformer and resnet hybrid model. Int. J. Adv. Res. Sci. Commun. Technol. 3, 734–743. doi: 10.48175/IJARSCT-12780

[ref23] Sabour S. Frosst N. Hinton G. E. (2017). Dynamic routing between capsules. Adv. Neural Inf. Process. Syst. 30.

[ref24] Sajitha N. Priya S. P. (2024). Optimal artificial neural network-based fabric defect detection and classification. Eng., Technol. Appl. Sci. Res. 14, 13148–13152. doi: 10.48084/etasr.6773

[ref25] Sastypratiwi H. Muhardi H. Yulianti Y. (2024). Batik recognition and classification using transfer learning and MobileNet approach. JOIV Int. J. Inform. Vis. 8, 2400–2410. doi: 10.62527/joiv.8.4.2407

[ref26] Selvaraju R. R. Cogswell M. Das A. Vedantam R. Parikh D. Batra D. (2020). Grad-CAM: visual explanations from deep networks via gradient-based localization. Int. J. Comput. Vis. 128, 336–359. doi: 10.1007/s11263-019-01228-7

[ref27] Shorten C. Khoshgoftaar T. M. (2019). A survey on image data augmentation for deep learning. J. Big Data 6, 1–48. doi: 10.1186/s40537-019-0197-0 PMC8287113 34306963

[ref28] Sun H. Lang W. Xu C. Liu N. Zhou H. (2023). Graph-based discriminative features learning for fine-grained image retrieval. Signal Process. Image Commun. 110:116885. doi: 10.1016/j.image.2022.116885

[ref29] Tan M. Le Q. (2021) EfficientNetV2: smaller models and faster training. In Proceedings of the 2021 International Conference on Machine Learning (pp. 10096–10106). Cambridge, MA: PMLR

[ref30] Tao P. Wenli C. Jia C. Xinghang L. Zili Z. Junping L. . (2023). Research on fabric classification based on graph neural network. Industria Textila 74, 3–11. doi: 10.35530/IT.074.01.202224

[ref31] Wang Y. Yao Q. Kwok J. T. Ni L. M. (2020). Generalizing from a few examples: a survey on few-shot learning. ACM Comput. Surv. 53, 1–34. doi: 10.1145/3386252

[ref32] Wei X. Hu B. Gao T. Wang J. Deng B. (2022). Multi-scale convolutional neural network for texture recognition. Displays 75:102324. doi: 10.1016/j.displa.2022.102324

[ref33] Woo S. Park J. Lee J. Y. Kweon I. S. (2018) CBAM: Convolutional block attention module. In Proceedings of the 2018 European Conference on Computer Vision (ECCV) (pp. 3–19). Cham: Springer

[ref34] Zhou X. Li H. Zhang D. (2023). Automatic fabric pattern recognition and design based on deep learning and portable device. Internet Technol. Lett. 6:e343. doi: 10.1002/itl2.343

